# Malnutrition Severity, Morphofunctional Assessment, and Survival in Patients with Lung Cancer: A Multicentre Prospective Cohort Study

**DOI:** 10.1007/s00408-026-00924-9

**Published:** 2026-08-14

**Authors:** Pablo Rodríguez de Vera-Gómez, María Galindo-Gallardo, María González-Pacheco, Alba Carmona-Llanos, María Teresa Zarco-Martín, Fiorella Ximena Palmas-Candia, Mª del Carmen Carmen Roque-Cuéllar, Araceli Muñoz-Garach, Concepción Muñoz-Jiménez, Mª Isabel Rebollo-Pérez, Vicente Mustieles, Rocío Fernández-Jiménez, María Luisa Fernández-Soto, José Manuel García-Almeida

**Affiliations:** 1 Department of Endocrinology and Nutrition, Virgen de la Macarena Universitary Hospital, 41009 Seville, Spain; 2 Fundación Pública Andaluza para la Gestión de la Investigación en Salud de Sevilla (FISEVI), Seville, Spain; 3 Instituto de Investigación e Innovación Biomédica de Cádiz (INiBICA), Hospital Universitario Puerta del Mar, Universidad de Cádiz, 11009 Cádiz, Spain; 4 Department of Endocrinology and Nutrition, Puerta del Mar University Hospital, 11009 Cádiz, Spain; 5 Clinical Medicine and Public Health, University of Granada, 18071 Granada, Spain; 6 Department of Endocrinology and Nutrition, Jerez Universitary Hospital, 11407 Jerez de la Frontera, Spain; 7 Department of Nutrition and Food Sciencie, University of Granada, 18071 Granada, Spain; 8 Endocrinology and Nutrition Unit, San Cecilio University Hospital, 18016 Granada, Spain; 9 Granada Biosanitary Research Institute (ibs.GRANADA), 18012 Granada, Spain; 10 Foundation for Biosanitary Research of Eastern Andalusia—Alejandro Otero (FIBAO), Granada, Spain; 11 Nutrition Support Unit, Endocrinology and Nutrition Department, Hospital Universitari Vall d’Hebron, 08035 Barcelona, Spain; 12 Endocrinología y Nutrición, Hospital Universitario Virgen del Rocío de Sevilla, 41013 Seville, Spain; 13 Department of Endocrinology and Nutrition, Virgen de las Nieves University Hospital, Granada, Spain; 14 CIBER Fisiopatología de la obesidad y Nutrición (CIBERObn), Instituto Salud Carlos III, Madrid, Spain; 15 Department of Endocrinology and Nutrition Reina, Sofía University Hospital, 14004 Cordoba, Spain; 16 Instituto Maimónides de Investigación Biomédica de Córdoba, 14004 Córdoba, Spain; 17 Department of Endocrinology and Nutrition Juan Ramón Jiménez University Hospital, 21005 Huelva, Spain; 18 Consortium for Biomedical Research in Epidemiology and Public Health (CIBERESP), Granada, Spain; 19 Servicio de Radiodiagnóstico Hospital Universitario Clínico San Cecilio, 18016 Granada, Spain; 20 Department of Endocrinology and Nutrition, Virgen de la Victoria University Hospital, 29010 Málaga, Spain; 21 IBIMA Plataforma BIONAND, Instituto de Investigación Biomédica de Málaga, Málaga, Spain; 22 Department of Endocrinology and Nutrition, Quironsalud Málaga Hospital, 29004 Málaga, Spain; 23 Department of Medicine and Dermatology, Faculty of Medicine, University of Málaga, 29016 Málaga, Spain; 24 Department of Medicine, University of Granada, 18016 Granada, Spain

**Keywords:** Lung cancer, Malnutrition, GLIM criteria, Bioelectrical impedance analysis, Muscle ultrasound, Morphofunctional assessment

## Abstract

**Purpose:**

Malnutrition is common in lung cancer and may contribute to impaired functional status and poor survival. This study evaluated the association between GLIM-defined malnutrition, morphofunctional nutritional parameters, and one-year mortality in patients with lung cancer.

**Methods:**

We conducted a multicentre prospective cohort study including 374 patients with primary lung cancer referred for nutritional and morphofunctional assessment before oncological treatment. Nutritional status was classified according to GLIM criteria. Body composition was assessed by bioelectrical impedance analysis, muscle ultrasound, handgrip strength and Timed Up and Go. Associations with severe malnutrition were explored using logistic regression, ROC analyses, and random forest models. One-year survival was evaluated using Kaplan–Meier curves and Cox regression models adjusted for age, sex, BMI, and ECOG performance status.

**Results:**

Severe malnutrition was present in 87 patients and was associated with lower albumin and prealbumin, higher C-reactive protein, poorer ECOG performance status, and higher one-year mortality. ECOG performance status worsened progressively across GLIM categories. Severe GLIM-defined malnutrition was associated with increased one-year mortality in unadjusted analysis (HR 4.31, 95% CI 2.73–6.80) and after adjustment for age, sex, BMI, and ECOG performance status (HR 3.32, 95% CI 1.96–5.61). Several morphofunctional markers, particularly phase angle, BCMI, BCM, FFMI, FFM, and ultrasound-derived rectus femoris parameters, showed discriminatory ability for severe malnutrition and exploratory associations with mortality.

**Conclusion:**

Severe GLIM-defined malnutrition is associated with poorer functional status and one-year mortality in lung cancer. Morphofunctional assessment may help characterize nutritional impairment, although proposed cut-offs require external validation.

**Supplementary Information:**

The online version contains supplementary material available at 10.1007/s00408-026-00924-9.

## Introduction

Lung cancer remains the leading cause of cancer-related mortality worldwide and is frequently accompanied by profound metabolic and functional impairment [1]. Beyond tumour burden, alterations in body composition and skeletal muscle function are important determinants of treatment tolerance, complications, quality of life, and survival [2–4]. These alterations are often under-recognized, even in early disease stages when muscle depletion and reduced physiological reserve may already be present despite preserved body weight [2–4]. Early nutritional and supportive interventions may improve clinical outcomes in this population [5, 6].

The Global Leadership Initiative on Malnutrition (GLIM) has established an internationally accepted framework for diagnosing malnutrition [7]. However, assessment of muscle mass within the GLIM criteria remains heterogeneous because of the wide range of techniques and cut-off values used across studies, potentially limiting diagnostic consistency and prognostic performance in patients with cancer [8–11].

Cancer-related malnutrition extends beyond weight loss and involves progressive alterations in muscle quantity, quality, and function. Accordingly, morphofunctional nutritional assessment has been proposed as an integrative approach combining body composition analysis with functional evaluation of skeletal muscle [12].

Among the available tools, bioelectrical impedance analysis (BIA) provides accessible measures of body composition and cellular integrity, including phase angle and body cell mass, with demonstrated prognostic value in oncology [13–17]. Rectus femoris ultrasound offers a reproducible assessment of muscle quantity associated with clinical outcomes [13, 18], while functional measures such as handgrip strength and the Timed Up and Go (TUG) test reflect muscle performance and have also been linked to survival and treatment tolerance [3, 19].

Despite these advances, evidence integrating morphofunctional assessment with malnutrition severity and prognosis in lung cancer remains limited, and its potential to improve risk stratification has not been fully established [20]. Therefore, we aimed to evaluate the relationship between nutritional status, body composition, muscle morphology, and muscle function in patients with primary lung cancer, and to determine whether morphofunctional parameters are associated with severe malnutrition and short-term mortality and may complement GLIM-based nutritional assessment [12, 20].

## Methods

### Study Design and Participants

This multicentre prospective cohort study with a cross-sectional baseline assessment was conducted within the VALONC study group, a Spanish collaborative network investigating the nutritional and morphofunctional impact of lung cancer, across 10 tertiary hospitals between 2023 and 2024. Participants underwent a standardized baseline assessment followed by prospective survival follow-up.

Adults (18–85 years) with confirmed primary lung cancer were eligible. Included patients were adults with primary lung cancer referred for nutritional and morphofunctional assessment before the initiation of an oncological treatment. In line with the real-world proposal of the study, inclusion was not restricted by disease stage, planned treatment modality, or treatment line. Therefore, patients could be assessed before first-line treatment or before subsequent treatment lines, including systemic therapy, radiotherapy, or surgery, according to clinical indication. Exclusion criteria were inability to complete the baseline assessment, estimated life expectancy < 3 months, or incomplete follow-up. Baseline evaluation was performed between the second and fourth week after diagnosis, before oncologic treatment or at the start of follow-up according to local clinical pathways. Vital status was recorded prospectively.

### Clinical, Anthropometric and Biochemical Assessment

Clinical data included age, sex, tumour histology, TNM stage, Eastern Cooperative Oncology Group (ECOG) performance status, and oncologic treatment. Weight, height, body mass index, and unintentional weight loss during the previous six months were recorded using standardized procedures.

Baseline laboratory assessment included serum albumin, prealbumin, creatinine, urea, total cholesterol, glycated haemoglobin (HbA1c), thyroid-stimulating hormone, 25-hydroxyvitamin D, and C-reactive protein.

### Body Composition, Muscle Ultrasound and Functional Assessment

Body composition was evaluated using the same phase-sensitive bioelectrical impedance analysis device across participating centres (BIA 101 BIVA PRO®, Akern Srl, Florence, Italy). Measurements were performed under standardized conditions using a tetrapolar hand-to-foot configuration, with electrodes placed on the ipsilateral wrist and ankle. Recorded and derived parameters included resistance, reactance, phase angle, standardized phase angle, fat mass, fat-free mass, body cell mass, fat-free mass index (FFMI), body cell mass index (BCMI), skeletal muscle index (SMI), and total and extracellular body water. BCMI was calculated as body cell mass divided by height squared.

Muscle morphology was assessed by rectus femoris ultrasound following a standardized protocol. Cross-sectional area, circumference, transverse and anteroposterior diameters, and subcutaneous and abdominal adipose tissue thickness were recorded.

Muscle function was assessed by handgrip strength using a calibrated dynamometer and the Timed Up and Go (TUG) test.

### Nutritional Assessment

Nutritional status was classified according to the Global Leadership Initiative on Malnutrition (GLIM) criteria [7], requiring at least one phenotypic and one etiologic criterion.

Muscle ultrasound was performed according to the standardized nutritional ultrasound protocol described by García-Almeida et al. [21]. Rectus femoris cross-sectional area and anteroposterior diameter were measured at predefined anatomical landmarks with the patient in a supine and relaxed position.

For the GLIM classification used in the survival analyses, severity was operationalized using predefined clinical and anthropometric phenotypic criteria, including BMI and unintentional weight loss, together with the etiologic criterion of active lung cancer. BIA- and ultrasound-derived muscle parameters were not used to define the GLIM categories analysed in the prognostic models; instead, they were evaluated as complementary morphofunctional markers. This approach was used to minimize direct circularity between the GLIM exposure and the morphofunctional predictors under evaluation. Applied thresholds and corresponding references are provided in the Supplementary Material. Patients were classified as well nourished, moderately malnourished, or severely malnourished.

### Statistical Analysis

Continuous variables were compared across GLIM categories using analysis of variance (ANOVA) or the Kruskal–Wallis test, according to distribution, while categorical variables were analysed using the chi-square test. Significant overall comparisons were followed by Bonferroni-adjusted pairwise analyses.

Associations between individual morphofunctional parameters and severe GLIM-defined malnutrition were evaluated using univariable logistic regression models, with one parameter entered per model. Variables were selected a priori based on their clinical relevance and their use in morphofunctional nutritional assessment. Results are expressed as odds ratios (ORs) with 95% confidence intervals (CIs). Because BIA-derived variables are mathematically and biologically interrelated, multicollinearity was expected. We therefore avoided simultaneous inclusion of multiple BIA parameters in the same regression model.

Optimal cut-off values were determined using the Youden index. These thresholds were derived within the study cohort and were subsequently evaluated as exploratory cut-offs; they were not internally or externally validated and were not used for GLIM classification. Sex-specific cut-offs were also explored in Cox models as prognostic thresholds.

A random forest analysis was performed as an exploratory approach to assess the relative importance of morphofunctional variables associated with severe GLIM-defined malnutrition. Severe malnutrition was used as the binary outcome, and candidate predictors included the morphofunctional parameters evaluated in the logistic regression analyses. The model was fitted using classification trees with bootstrap aggregation, and variable importance was ranked according to mean decrease in Gini impurity.

Survival was analysed using Kaplan–Meier curves, log-rank tests, and Cox proportional hazards models. For GLIM-defined nutritional status, unadjusted and multivariable-adjusted Cox models were fitted, with adjustment for age, sex, body mass index (BMI), and Eastern Cooperative Oncology Group (ECOG) performance status. ECOG performance status was compared across GLIM-defined nutritional categories using the Kruskal–Wallis test, with pairwise Wilcoxon rank-sum tests and Holm correction. Associations between ECOG performance status and morphofunctional parameters were explored using Spearman correlation coefficients, given the ordinal nature of ECOG.

Morphofunctional cut-offs derived from sex-stratified ROC analyses were subsequently evaluated as exploratory prognostic thresholds using sex-stratified Cox models adjusted for age, BMI, and ECOG performance status. Results are reported as hazard ratios (HRs) with 95% confidence intervals (CIs). The proportional hazards assumption was assessed using Schoenfeld residuals. Missing data were assessed for the main variables included in the survival and morphofunctional analyses and are reported in Supplementary Material. Analyses were performed using a complete-case approach for each model.

Statistical significance was defined as *p* < 0.05. Analyses were performed using R software (R Foundation for Statistical Computing, Vienna, Austria).

### Ethics

The study complied with the Declaration of Helsinki and applicable national regulations. Ethical approval was obtained before study initiation (25 July 2023; protocol 1085-N-23), and all participants provided written informed consent.

## Results

### Baseline Characteristics

A total of 374 patients with primary lung cancer were included and categorized according to nutritional status as well-nourished (n = 198), moderately malnourished (n = 89), and severely malnourished (n = 87) (Table 1).

**Table 1:** Summary descriptive table of the patients included in the study, divided by GLIM criteria

Variables	All *N* = *374*	Well *N* = *198*	Moderate *N* = *89*	Severe *N* = *87*	*P* value
Age (years)	66.8 (9.65)	66.1 (9.38)	67.3 (9.87)	67.8 (10.0)	0.342
*Sex*					0.192
Females	114 (30.6%)	54 (27.6%)	34 (38.2%)	26 (29.9%)	
Males	258 (69.4%)	142 (72.4%)	55 (61.8%)	61 (70.1%)	
BMI, kg/m^2^	26.1 (4.93)	28.3 (4.07)	24.5 (4.25)	22.8 (4.93)	< 0.001
HbA1c (%)	6.25 (0.90)	6.24 (0.92)	6.18 (0.91)	6.31 (0.85)	0.630
Total cholesterol (mg/dL)	180 (38.0)	183 (40.6)	179 (33.7)	174 (35.4)	0.139
Urea (mg/dL)	41.6 (18.7)	41.8 (17.0)	40.4 (18.9)	42.4 (22.2)	0.789
Creatinine (mg/dL)	0.95 (0.77)	1.00 (0.89)	0.92 (0.54)	0.89 (0.66)	0.498
Glomerular filtration (mL/min/1.73 m^2^)	81.9 (20.2)	81.2 (21.3)	80.8 (17.1)	84.4 (20.8)	0.400
Proteins (mg/dL)	6.97 (0.58)	7.01 (0.53)	7.00 (0.54)	6.86 (0.69)	0.191
Prealbumin (mg/dL)	23.3 (5.88)	24.3 (5.04)	23.0 (7.22)	21.5 (5.75)	0.001
Albumin (mg/dL)	3.96 (0.57)	4.07 (0.57)	3.83 (0.57)	3.85 (0.52)	< 0.001
C reactive protein (mg/dL)	35.2 (59.4)	27.8 (39.8)	40.7 (91.0)	46.5 (54.2)	0.011
TSH (μUI/mL)	1.83 (1.11)	1.78 (0.86)	2.10 (1.65)	1.69 (0.88)	0.130
*ECOG*					< 0.001
0	153 (41.4%)	107 (54.3%)	30 (33.7%)	16 (19.0%)	
1	153 (41.4%)	77 (39.1%)	42 (47.2%)	34 (40.5%)	
2	53 (14.3%)	10 (5.08%)	13 (14.6%)	30 (35.7%)	
3–4	11 (2.97%)	3 (1.52%)	4 (4.49%)	4 (4.76%)	
*Cancer type*					0.267
NSCLC	283 (76.1%)	147 (74.2%)	69 (78.4%)	67 (77.9%)	
Other	13 (3.49%)	10 (5.05%)	3 (3.41%)	0 (0.00%)	
SCLC	30 (8.06%)	14 (7.07%)	7 (7.95%)	9 (10.5%)	
Mesothelioma	9 (2.42%)	3 (1.52%)	3 (3.41%)	3 (3.49%)	
Not specified	37 (9.95%)	24 (12.1%)	6 (6.82%)	7 (8.14%)	
*TNM*					< 0.001
I	29 (11.9%)	19 (15.0%)	5 (8.47%)	5 (8.62%)	
II	18 (7.38%)	12 (9.45%)	4 (6.78%)	2 (3.45%)	
III	25 (10.2%)	16 (12.6%)	8 (13.6%)	1 (1.72%)	
IVa	70 (28.7%)	39 (30.7%)	15 (25.4%)	16 (27.6%)	
IVb	25 (10.2%)	13 (10.2%)	7 (11.9%)	5 (8.62%)	
IVc	77 (31.6%)	28 (22.0%)	20 (33.9%)	29 (50.0%)	
*Chemotherapy*					0.014
No	140 (40.1%)	81 (44.8%)	36 (43.9%)	23 (26.7%)	
Yes	209 (59.9%)	100 (55.2%)	46 (56.1%)	63 (73.3%)	
*Radiotherapy*					0.261
No	212 (60.4%)	118 (64.5%)	46 (55.4%)	48 (56.5%)	
Yes	139 (39.6%)	65 (35.5%)	37 (44.6%)	37 (43.5%)	
*Immunotherapy*					0.018
No	277 (80.5%)	152 (84.4%)	67 (82.7%)	58 (69.9%)	
Yes	67 (19.5%)	28 (15.6%)	14 (17.3%)	25 (30.1%)	
*Complications*					< 0.001
Absence	216 (57.8%)	141 (71.2%)	41 (46.1%)	34 (39.1%)	
Presence	158 (42.2%)	57 (28.8%)	48 (53.9%)	53 (60.9%)	
*Exitus*					< 0.001
No	238 (63.6%)	146 (73.7%)	56 (62.9%)	36 (41.4%)	
Yes	136 (36.4%)	52 (26.3%)	33 (37.1%)	51 (58.6%)	

Data are shown as mean (SD) or n (%). Patients were grouped according to GLIM-defined nutritional status. Normality was assessed using the Shapiro–Wilk test. Between-group comparisons were performed using ANOVA or the Kruskal–Wallis test for continuous variables, and the chi-square or Fisher exact test for categorical variables, as appropriate. Post-hoc pairwise comparisons were corrected for multiple testing

BMI, body mass index; CRP, C-reactive protein; ECOG, eastern cooperative oncology group; GLIM, global leadership initiative on malnutrition; HbA1c, glycated hemoglobin; NSCLC, non-small cell lung cancer; SCLC, small-cell lung cancer; SD, standard deviation; TNM, tumour-node-metastasis; TSH, thyroid-stimulating hormone

Patients with moderate and severe malnutrition had significantly lower body mass index compared with well-nourished individuals (*p* < 0.001). Markers of nutritional status were progressively impaired across groups, with lower serum albumin and prealbumin levels in the severe malnutrition group (*p* < 0.001 and *p* = 0.001, respectively), alongside higher C-reactive protein levels (*p* = 0.011).

Functional status also differed significantly, with a higher proportion of patients with ECOG ≥ 2 in the moderate and severe malnutrition categories. ECOG performance status worsened progressively across GLIM-defined nutritional categories (*p* < 0.001). Patients with severe malnutrition had higher ECOG scores than both well-nourished and moderately malnourished patients (Holm-adjusted *p* < 0.001 and *p* = 0.003, respectively). In exploratory correlation analyses, worse ECOG was most strongly associated with higher Timed Up and Go values and lower phase angle, BCMI, BCM, RF-Y-axis, and RF-CSA (all *p* < 0.001) (Supplementary Material).

Patients with severe malnutrition were more likely to receive chemotherapy (*p* = 0.014) and immunotherapy (*p* = 0.018), and had a higher frequency of complications (*p* < 0.001). Mortality increased progressively across GLIM categories, reaching 58.6% in the severe malnutrition group compared with 37.1% in moderate and 26.3% in well-nourished patients (*p* < 0.001) (Table 1). Detailed pairwise comparisons are provided in the Supplementary Material.

### Morphofunctional Assessment Across GLIM Categories

Morphofunctional parameters showed a consistent and progressive deterioration across GLIM-defined nutritional categories (Table 2). Patients with moderate and severe malnutrition presented lower body weight (79.2 vs. 67.8 vs. 62.3 kg, *p* < 0.001) and greater unintentional weight loss (− 0.24% vs. 6.23% vs. 17.4%, *p* < 0.001). Bioelectrical impedance parameters demonstrated marked alterations, including reductions in phase angle (5.28 vs. 4.93 vs. 4.63°, *p* < 0.001), fat-free mass index (19.7 vs. 17.9 vs. 17.3 kg/m^2^, *p* < 0.001), and body cell mass index (9.82 vs. 8.55 vs. 7.93 kg/m^2^, *p* < 0.001).

**Table 2:** Summary descriptive table of BIVA and ultrasound measurement measured in the patients included in this study and divided by GLIM criteria

Variables	All *N = 374*	Well *N = 198*	Moderate *N = 89*	Severe *N = 87*	*P* value
*Anthropometric*					
Weight, kg	72.6 (15.9)	79.2 (13.9)	67.8 (14.0)	62.3 (14.8)	< 0.001
Weight loss	5.41 (10.2)	− 0.24 (3.50)	6.23 (3.33)	17.4 (13.9)	< 0.001
*BIVA variables*					
Xc	47.1 (9.32)	47.1 (8.85)	48.1 (8.92)	45.9 (10.7)	0.343
Rz	535 (85.3)	510 (71.8)	560 (89.5)	568 (91.2)	< 0.001
PA (°)	5.05 (0.80)	5.28 (0.70)	4.93 (0.69)	4.63 (0.92)	< 0.001
SPA	− 0.58 (1.17)	− 0.37 (1.04)	− 0.53 (1.28)	− 1.10 (1.19)	< 0.001
BCM, kg	25.2 (5.88)	27.5 (5.43)	23.7 (4.95)	21.7 (5.53)	< 0.001
FM, kg	20.4 (9.43)	23.7 (8.54)	18.1 (9.34)	14.9 (8.29)	< 0.001
FFMI, kg/m^2^	18.7 (2.45)	19.7 (2.16)	17.9 (2.09)	17.3 (2.42)	< 0.001
FMI, kg/m^2^	7.32 (3.39)	8.50 (3.09)	6.55 (3.38)	5.46 (3.00)	< 0.001
BCMI, kg/m^2^	9.07 (1.75)	9.82 (1.51)	8.55 (1.39)	7.93 (1.79)	< 0.001
SMI, kg/m^2^	8.77 (1.57)	9.16 (1.50)	8.31 (1.64)	8.37 (1.47)	< 0.001
MM, kg	27.7 (6.83)	29.5 (7.19)	26.5 (6.42)	24.8 (4.95)	< 0.001
ASMM, kg	19.5 (4.56)	21.0 (4.67)	18.3 (3.95)	17.4 (3.59)	< 0.001
FFM, kg	52.1 (9.15)	55.3 (8.76)	49.6 (8.25)	47.3 (8.04)	< 0.001
Hydragram®, %	74.3 (2.55)	74.2 (2.39)	74.1 (2.09)	74.6 (3.24)	0.406
Nutrigram®, mg/24 h/m	758 (178)	819 (174)	713 (142)	664 (168)	< 0.001
Basal metabolism	1455 (263)	1516 (269)	1423 (209)	1348 (262)	< 0.001
TBW, kg	38.7 (7.10)	41.1 (6.81)	36.9 (6.71)	35.4 (6.22)	< 0.001
ECW, kg	19.7 (3.67)	20.3 (3.71)	18.9 (3.60)	18.8 (3.37)	0.001
Na/K	1.18 (0.26)	1.10 (0.23)	1.20 (0.19)	1.34 (0.30)	< 0.001
*Ultrasound variables*					
RF-CSA, cm^2^	3.89 (1.36)	4.37 (1.22)	3.48 (1.20)	3.23 (1.41)	< 0.001
RF-CIRC, cm	9.08 (1.27)	9.44 (1.08)	8.80 (1.25)	8.57 (1.43)	< 0.001
RF-X-axis, cm	3.74 (0.82)	3.88 (0.87)	3.60 (0.54)	3.54 (0.89)	0.001
RF-Y-axis, cm	1.15 (0.36)	1.28 (0.32)	1.04 (0.30)	0.96 (0.40)	< 0.001
L-SAT, cm	0.80 (0.45)	0.89 (0.49)	0.74 (0.41)	0.66 (0.34)	< 0.001
T-SAT, cm	1.46 (0.66)	1.60 (0.68)	1.46 (0.65)	1.13 (0.51)	< 0.001
S-SAT, cm	0.64 (0.36)	0.69 (0.37)	0.65 (0.37)	0.53 (0.28)	< 0.001
VAT, cm	0.60 (0.36)	0.68 (0.38)	0.51 (0.26)	0.50 (0.35)	< 0.001
GAT, cm	2.85 (1.10)	3.17 (1.10)	2.71 (1.07)	2.29 (0.87)	< 0.001
GATi, cm/m^2^	0.02 (0.01)	0.02 (0.01)	0.02 (0.01)	0.01 (0.01)	< 0.001
*Functional variables*					
Hand grip strength max, kg	31.4 (10.2)	33.8 (9.91)	29.9 (9.93)	27.4 (9.55)	< 0.001
Hand grip strength, kg	29.5 (10.1)	31.9 (9.84)	28.0 (9.77)	25.4 (9.61)	< 0.001
Up and Go, s	8.94 (3.36)	8.13 (2.56)	9.09 (3.24)	10.6 (4.36)	< 0.001

Data are represented as mean (SD) or n (%). Groups were divided according to the GLIM criteria, being well, moderate and severe the categories included in the study. A Shapiro-Wilks test was used to determine the normality of the variables

BCM, body cell mass; BCMI, BCM index; BMI, body mass index; BIVA, bioelectrical impedance vectorial analysis; FM, fat mass; FMI, FM index; FFMI, fat-free mass index; GLIM, the global leadership initiative on malnutrition; PA, phase angle; RF-CIR, circumference of quadriceps rectus femoris; RF-CSA, rectus femoris cross-sectional area; SAT, subcutaneous adipose fat of leg (L), superficial (S) and total (T) abdominal; SMI, skeletal muscle index; SPA, standardized phase angle

Ultrasound measurements confirmed reduced muscle quantity, with lower rectus femoris cross-sectional area (4.37 vs. 3.48 vs. 3.23 cm^2^, *p* < 0.001), together with decreases in muscle thickness and circumference. Adipose tissue compartments were also reduced in the severe group (all *p* < 0.001).

Functional performance was impaired in parallel, with reduced handgrip strength (33.8 vs. 29.9 vs. 27.4 kg, *p* < 0.001) and longer Timed Up and Go times (8.13 vs. 9.09 vs. 10.6 s, *p* < 0.001). Detailed pairwise comparisons are provided in the Supplementary Material.

### Association Between Morphofunctional Parameters and Severe Malnutrition

Logistic regression analysis comparing well-nourished and severely malnourished patients identified multiple morphofunctional parameters associated with severe GLIM-defined malnutrition (Table 3).

**Table 3:** Odds ratios for severe GLIM status (well vs. severe) according to BIVA, ultrasound, handgrip strength, and functional performance

Variables	OR (CI 95%)	*P* value
*BIVA variables*		
PA (°)	0.54 (0.35–0.82)	0.005
SPA	0.86 (0.67–1.1)	0.237
BCM, kg	0.9 (0.83–0.98)	0.019
FM, kg	1.08 (1.01–1.17)	0.025
FFMI, kg/m^2^	0.76 (0.6–0.95)	0.020
FMI, kg/m^2^	1.31 (1.04–1.67)	0.024
BCMI, kg/m^2^	0.67 (0.51–0.86)	0.003
SMI, kg/m^2^	0.82 (0.62–1.07)	0.149
MM, kg	0.98 (0.93–1.04)	0.572
ASMM, kg	0.93 (0.82–1.02)	0.187
FFM, kg	0.96 (0.9–1.02)	0.218
TBW, kg	0.97 (0.9–1.04)	0.393
ECW, kg	1.05 (0.94–1.17)	0.415
Na/K	1.88 (0.5–9.09)	0.387
*Ultrasound*		
RF-CSA, cm^2^	0.61 (0.45–0.82)	0.001
RF-CIRC, cm	0.69 (0.52–0.91)	0.008
RF-X-axis, cm	0.51 (0.28–0.89)	0.023
RF-Y-axis, cm	0.2 (0.06–0.59)	0.005
L-SAT, cm	0.82 (0.33–1.96)	0.654
T-SAT, cm	1.3 (0.76–2.24)	0.336
S-SAT, cm	2.16 (0.82–5.5)	0.110
VAT, cm	0.22 (0.07–0.59)	0.005
GAT, cm	0.87 (0.6–1.23)	0.435
*Functional variables*		
Hand grip strength max, kg	0.98 (0.95–1.02)	0.321
Hand grip strength, kg	0.98 (0.94–1.01)	0.237
Up and Go, s	1.18 (1.06–1.32)	0.003

This table shows the results of logistic regression analysis assessing the association between GLIM nutritional status (**well** vs. **severe**) and various clinical and functional variables. The dependent variable is GLIM status, with the **well** group as the reference and **severe** as the outcome of interest. Independent variables include measurements from bioelectrical impedance analysis (BIVA), ultrasound parameters, handgrip strength, and functional performance assessed by the **Timed Up and Go** test. For each variable, odds ratios (OR) with 95% confidence intervals (CI) and *p*-values are reported. The ORs represent the change in odds of being classified as severe GLIM per unit increase (or category) of the respective variable. An OR > 1 indicates higher odds of severe GLIM with increasing values, whereas an OR < 1 indicates lower odds

BCM, body cell mass; BCMI, BCM index; BMI, body mass index; BIVA, bioelectrical impedance vectorial analysis; FM, fat mass; FMI, FM index; FFMI, fat-free mass index; GLIM, the global leadership initiative on malnutrition; PA, phase angle; RF-CIR, circumference of quadriceps rectus femoris; RF-CSA, rectus femoris cross-sectional area; SAT, subcutaneous adipose fat of leg (L), superficial (S) and total (T) abdominal; SMI, skeletal muscle index; SPA, standardized phase angle

Among BIA-derived variables, lower phase angle (OR 0.54, 95% CI 0.35–0.82, *p* = 0.005), fat-free mass index (OR 0.76, 95% CI 0.60–0.95, *p* = 0.020), and body cell mass index (OR 0.67, 95% CI 0.51–0.86, *p* = 0.003) were significantly associated with severe malnutrition. Body cell mass also showed a significant association (OR 0.90, 95% CI 0.83–0.98, *p* = 0.019). In contrast, higher fat mass index was associated with increased odds of severe malnutrition (OR 1.31, 95% CI 1.04–1.67, *p* = 0.024).

Ultrasound-derived parameters, including rectus femoris cross-sectional area (OR 0.61, *p* = 0.001) and muscle circumference (OR 0.69, *p* = 0.008), were also significantly associated with severe malnutrition. Among functional measures, longer Timed Up and Go time was associated with higher odds of severe malnutrition (OR 1.18, 95% CI 1.06–1.32, *p* = 0.003), whereas handgrip strength was not statistically significant. Additional analyses are provided in the Supplementary Material.

### Variable Importance and Discriminative Performance

**Fig. 1:**
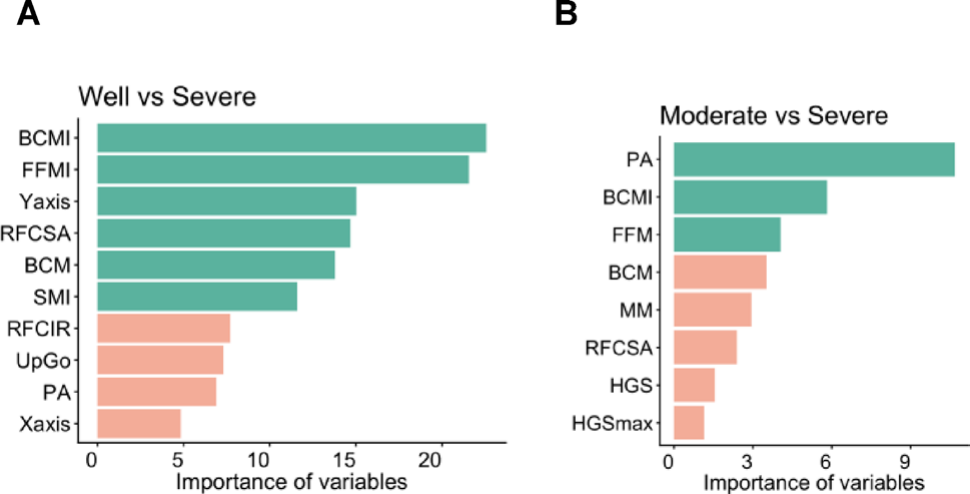
Variable importance of morphofunctional biomarkers for the identification of severe malnutrition in patients with lung cancer. Variable importance rankings derived from Random Forest models evaluating the contribution of morphofunctional parameters to the identification of severe GLIM-defined malnutrition. **A** Comparison between well-nourished and severely malnourished patients. Body cell mass index (BCMI), fat-free mass index (FFMI), rectus femoris anteroposterior diameter (RF Y-axis), and rectus femoris cross-sectional area (RFCSA) were the most relevant predictors. **B** Comparison between moderately and severely malnourished patients. Phase angle (PA), BCMI, and fat-free mass (FFM) showed the highest discriminative importance. Variable importance was quantified using the mean decrease in Gini index. Higher values indicate a greater contribution of the variable to classification performance. *Abbreviations* BCM, body cell mass; BCMI, body cell mass index; FFM, fat-free mass; FFMI, fat free mass index; HGS, handgrip strength; HGSmax, maximum handgrip strength; MM, muscle mass; PA, phase angle; RFCIR, rectus femoris circumference; RFCSA, rectus femoris cross sectional area; Xaxis, rectus femoris transverse diameter (X-axis); Yaxis, rectus femoris anteroposterior diameter (Y-axis); UpGo, timed up and go test

A random forest analysis was performed to assess the relative importance of morphofunctional variables in identifying severe malnutrition. In the comparison between well-nourished and severely malnourished patients, fat-free mass index, rectus femoris cross-sectional area, body cell mass, body cell mass index, skeletal muscle index, and rectus femoris Y-axis emerged as the most relevant variables (Fig. 1A).

When comparing moderate and severe malnutrition, phase angle, body cell mass, and fat-free mass showed the highest relative importance (Fig. 1B).

Receiver operating characteristic (ROC) analyses confirmed the discriminative capacity of the selected morphofunctional parameters for identifying severe malnutrition. Rectus femoris cross-sectional area showed the highest performance, particularly in women (AUC 0.87), followed by body cell mass, body cell mass index, and fat-free mass index. Detailed ROC results and sex-specific cut-off values are provided in Table 4.

**Table 4:** Discriminative performance of muscle and body composition parameters for detecting severe malnutrition and sex-stratified adjusted association with one-year mortality

Variables	Sex	Cut-off, unit	AUC	Sensitivity	Specificity	*P* value for AUC	Adjusted HR for mortality (95% CI)	*P* value for mortality
RF-CSA	Men	3.48 cm^2^	0.727	0.838	0.574	< 0.001	1.45 (0.83–2.51)	0.188
	Women	2.97 cm^2^	0.87	0.815	0.846	< 0.001	0.78 (0.25–2.49)	0.678
BCM	Men	23.50 kg	0.79	0.937	0.574	< 0.001	1.93 (1.06–3.52)	0.031
	Women	18.50 kg	0.859	0.926	0.692	< 0.001	1.21 (0.44–3.33)	0.716
BCMI	Men	8.37 kg/m^2^	0.782	0.908	0.573	< 0.001	2.66 (1.49–4.72)	< 0.001
	Women	7.34 kg/m^2^	0.852	0.731	0.852	< 0.001	1.91 (0.65–5.55)	0.237
SMI	Men	8.80 kg/m^2^	0.714	0.782	0.59	< 0.001	1.56 (0.89–2.73)	0.12
	Women	7.20 kg/m^2^	0.623	0.684	0.615	0.074	1.88 (0.71–5.01)	0.205
FFM	Men	54.20 kg	0.792	0.738	0.738	< 0.001	1.86 (1.00–3.47)	0.049
	Women	43.20 kg	0.72	0.769	0.623	0.001	1.86 (0.65–5.35)	0.249
FFMI	Men	18.01 kg/m^2^	0.795	0.908	0.59	< 0.001	2.31 (1.22–4.36)	0.01
	Women	16.78 kg/m^2^	0.792	0.815	0.769	< 0.001	0.72 (0.20–2.53)	0.609
PA	Men	4.75°	0.7	0.508	0.809	< 0.001	1.93 (1.09–3.42)	0.025
	Women	4.75°	0.789	0.731	0.82	< 0.001	1.98 (0.72–5.41)	0.183
RF-Y-axis	Men	0.91 cm	0.741	0.936	0.492	< 0.001	1.57 (0.86–2.85)	0.143
	Women	0.86 cm	0.834	0.926	0.615	< 0.001	0.93 (0.29–3.00)	0.898

AUC, cut-off values, sensitivity, specificity, and *p*-values refer to the discrimination between well-nourished and severely malnourished patients. Adjusted hazard ratios (HRs) for one-year mortality were estimated using sex-stratified Cox regression models adjusted for age, BMI, and ECOG performance status. HRs represent the mortality risk associated with values below the sex-specific cut-off. For BIA-derived markers, 242 men and 113 women were included; for RF-CSA, 236 men and 106 women; and for RF-Y-axis, 233 men and 105 women. CI, confidence interval; AUC, area under the curve; BCM, body cell mass; BCMI, body cell mass index; ECOG, eastern cooperative oncology group; FFM, fat-free mass; FFMI, fat-free mass index; PA, phase angle; RF-CSA, rectus femoris cross-sectional area; RF-Y-axis, rectus femoris anteroposterior diameter; SMI, skeletal muscle index

Discriminative capacity was lower when comparing moderate versus severe malnutrition, although phase angle and body cell mass index retained moderate performance, especially in women (AUC up to 0.72).

### Survival Analysis

**Fig. 2:**
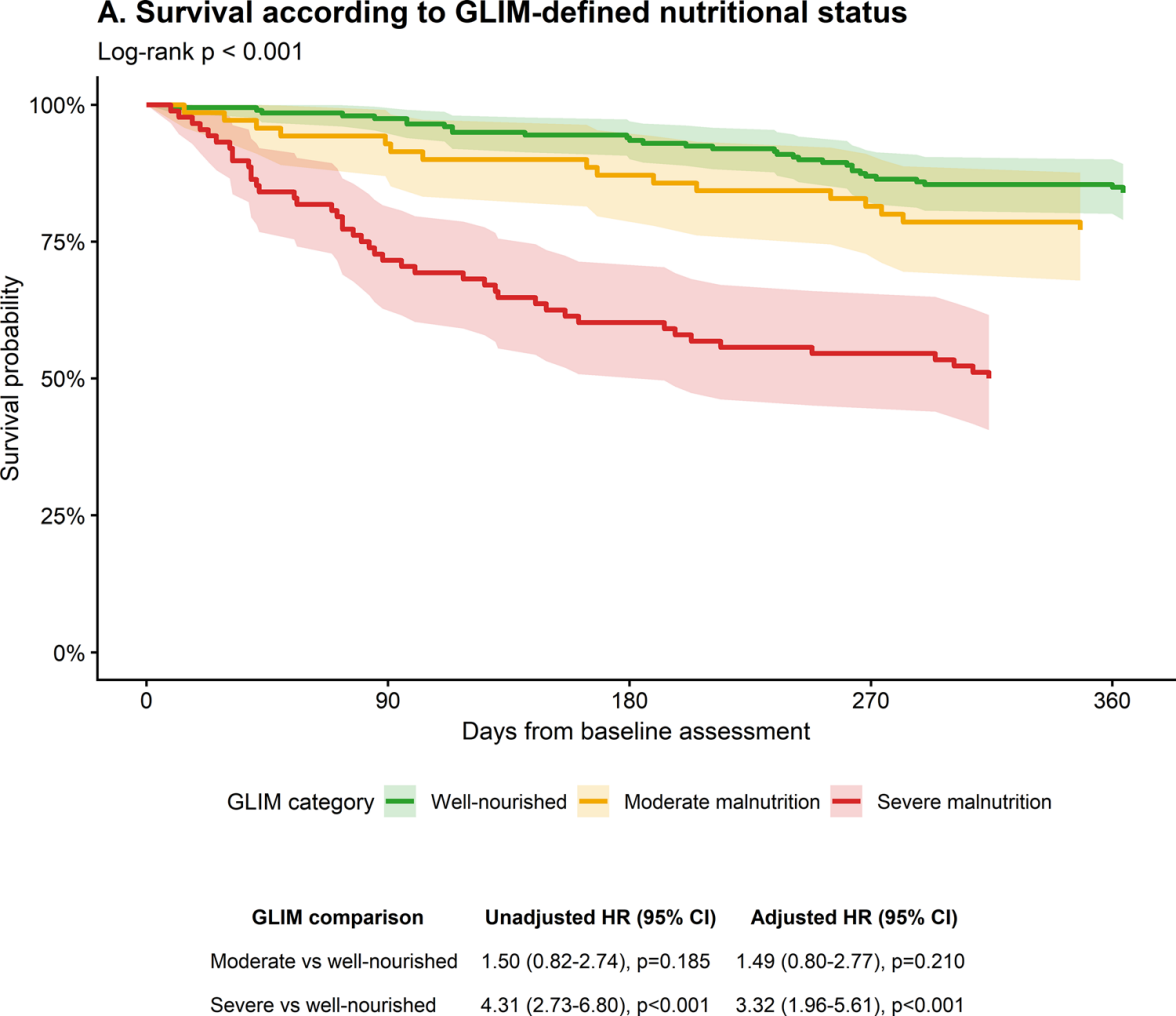
One-year survival according to GLIM-defined nutritional status. Kaplan–Meier curves showing one-year overall survival according to GLIM nutritional category. Shaded areas represent 95% confidence intervals. The table below the curves shows unadjusted and adjusted hazard ratios (HRs) with 95% confidence intervals for moderate and severe malnutrition, using well-nourished patients as the reference group. Adjusted HRs were estimated using Cox proportional hazards models adjusted for age, sex, body mass index, and ECOG performance status. GLIM, Global Leadership Initiative on Malnutrition; ECOG, Eastern Cooperative Oncology Group; HR, hazard ratio; CI, confidence interval

During the 12-month follow-up period, 136 deaths (36.4%) were recorded. Kaplan–Meier analysis showed progressively reduced survival across GLIM categories, with the lowest survival among patients with severe malnutrition (log-rank *p* < 0.001) (Fig. 2).

In Cox regression, severe GLIM-defined malnutrition was associated with increased one-year mortality in the unadjusted analysis (HR 4.31, 95% CI 2.73–6.80, *p* < 0.001) and remained associated after adjustment for age, sex, BMI, and ECOG performance status (HR 3.32, 95% CI 1.96–5.61, *p* < 0.001). Moderate malnutrition was not significantly associated with mortality in either unadjusted (HR 1.50, 95% CI 0.82–2.74, *p* = 0.185) or adjusted analysis (HR 1.49, 95% CI 0.80–2.77, *p* = 0.210). Schoenfeld residual testing suggested some evidence of non-proportionality for GLIM-defined nutritional status, whereas the global test for the adjusted model was borderline and did not reach conventional statistical significance (*p* = 0.062, Supplementary Material).

Sex-specific morphofunctional cut-offs were also evaluated for their association with one-year mortality (Table 4). In men, low BCMI, BCM, FFMI, FFM, and phase angle were associated with increased mortality after adjustment for age, BMI, and ECOG performance status. In women, effect estimates were less precise, with wider confidence intervals, consistent with the smaller number of events.

## Discussion

In this multicentre cohort of patients with lung cancer, morphofunctional biomarkers were strongly associated with both malnutrition severity and mortality, suggesting that they improve prognostic stratification beyond conventional nutritional assessment. These findings reinforce the growing recognition that muscle-related alterations represent a major component of cancer vulnerability beyond weight loss alone [2–4, 22, 23].

The progressive decline in morphofunctional parameters across GLIM categories confirms that cancer-related malnutrition extends beyond weight loss and reflects depletion of metabolically active tissue, muscle wasting, and functional impairment [2–4, 22, 23]. Among the evaluated parameters, bioelectrical impedance–derived indices, particularly body cell mass index (BCMI) and fat-free mass index (FFMI), showed strong associations with malnutrition severity, supporting previous evidence that BIA provides valuable information on cellular integrity and nutritional risk in oncology [13–16, 24]. Recent studies further suggest that the prognostic value of BIA extends beyond phase angle alone [15, 16].

Phase angle, a surrogate of cell membrane integrity, declined progressively across GLIM categories, with values consistent with advanced physiological impairment. These findings are lower than those reported in healthy populations [25] and agree with previous oncology studies linking reduced phase angle to poorer prognosis [14–17, 26], supporting its role as a marker of adverse clinical outcomes [17, 24, 27]. In lung cancer, our results further reinforce its value within a broader morphofunctional assessment [17].

Muscle ultrasound provided complementary information. Progressive reductions in rectus femoris cross-sectional area and muscle thickness were consistent with previous reports of muscle depletion in cancer [18, 28–30]. Given its feasibility, reproducibility, and bedside applicability, quadriceps ultrasound may enhance phenotypic assessment of malnutrition, particularly where more complex imaging techniques are unavailable [18, 28, 29, 31]. Recent ultrasound-based thresholds further support its role in nutritional and sarcopenia-oriented assessment [31].

Together, these findings indicate that integrating body composition and muscle function with conventional nutritional assessment provides a more comprehensive evaluation of vulnerability in lung cancer [12], particularly in patients with cachexia and systemic inflammation, where clinically relevant muscle depletion may occur despite preserved body weight or body mass index [3, 22, 23, 32].

Morphofunctional parameters also showed prognostic associations when evaluated using predefined sex-specific cut-offs, although these analyses should be considered exploratory rather than definitive evidence of incremental prognostic value beyond established clinical models [9–11]. This agrees with previous reports linking poor nutritional status and morphofunctional impairment to adverse outcomes in lung cancer and other solid tumours [4, 19, 28, 29, 32].

BCMI emerged as the most robust predictor of mortality across both sexes, a finding that is biologically plausible because body cell mass reflects metabolically active tissue and physiological reserve [24]. This observation supports growing evidence that BIA-derived variables beyond phase angle have prognostic relevance in oncology [15, 16]. In contrast, FFMI was more strongly associated with mortality in men, whereas phase angle showed greater prognostic value in women, possibly reflecting known sex-related differences in body composition and fluid distribution [14, 16, 26].

The association between higher fat mass index and severe malnutrition may reflect sarcopenic obesity, in which excess adiposity coexists with reduced muscle mass [28, 32]. This finding highlights the limitations of relying exclusively on weight- or fat-based measures and reinforces the value of morphofunctional assessment for identifying qualitative changes in skeletal muscle [12, 22, 23].

An additional exploratory finding of this study is the description of cohort-derived, sex-specific cut-off values for selected morphofunctional parameters. These thresholds showed discriminatory performance for severe GLIM-defined malnutrition and were associated with one-year mortality for some markers, particularly in men. Their differences from conventional GLIM thresholds suggest that earlier alterations in muscle mass and cellular integrity may already have prognostic significance in lung cancer, supporting the development of disease-specific thresholds [7, 10, 11, 20, 31]. These findings are consistent with previous studies combining GLIM and morphofunctional assessment [11, 20, 30]. However, because they were derived and evaluated within the same cohort, they should be interpreted as hypothesis-generating rather than clinically validated thresholds.

Clinically, our findings support incorporating morphofunctional assessment into the routine evaluation of patients with lung cancer [12, 13, 16]. BCMI and phase angle emerged as accessible markers associated with mortality risk [14–17, 24], while integrating nutritional and muscle-related parameters may facilitate earlier supportive care, nutritional intervention, and closer clinical monitoring [3–5, 10, 32, 33].

This study has several strengths, including its multicentre prospective design, relatively large cohort, comprehensive morphofunctional assessment, and complementary analytical approach. Nevertheless, some limitations should be acknowledged. First, given the observational and real-world design, residual confounding and clinical heterogeneity cannot be excluded. Patients were included before different oncological treatment strategies and were not restricted to a single disease stage or treatment line. Although survival models were adjusted for age, sex, BMI, and ECOG performance status, information on TNM stage, treatment modality, inflammatory status, molecular tumour profiling, and treatment response was incomplete or not systematically available. Therefore, survival findings should be interpreted as adjusted associations rather than evidence of independent causal effects or incremental prognostic value beyond established oncological models. In addition, the proportional hazards assumption was not fully satisfied for GLIM-defined nutritional status, suggesting that the mortality risk associated with malnutrition may vary during follow-up; Cox estimates should therefore be interpreted as average associations over the one-year period.

Second, although BIA- and ultrasound-derived muscle parameters were not used to define the GLIM categories analysed in the survival models, these markers remain conceptually related to the reduced muscle mass domain of the GLIM framework. Accordingly, analyses evaluating their discrimination of severe GLIM-defined malnutrition should be considered exploratory and may be influenced by conceptual overlap. Formal sarcopenia diagnosis according to established criteria was not performed; instead, individual morphofunctional components related to muscle mass, morphology, and function were analysed separately. Because BIA-derived variables represent related body-composition compartments, multivariable diagnostic models including several BIA parameters simultaneously were not fitted, and logistic regression results should be interpreted as marker-specific associations. Similarly, the ROC-derived cut-off values and random forest analysis were derived and evaluated within the same cohort, without internal or external validation, and should be considered hypothesis-generating rather than clinically validated tools. Finally, although post-hoc pairwise comparisons were corrected for multiple testing, the large number of secondary analyses may have increased the risk of type I error. Muscle ultrasound was performed according to a standardized published protocol, but formal interobserver variability was not assessed, and longitudinal changes in body composition were not evaluated.

In summary, malnutrition and morphofunctional impairment are common and clinically relevant in lung cancer. Integrating body composition and muscle function into nutritional assessment improves risk stratification and may facilitate earlier identification of patients at increased risk of short-term mortality. Parameters reflecting cellular integrity, particularly BCMI and phase angle, may provide useful prognostic information and help guide supportive and nutritional strategies in this high-risk population.

## Supplementary Information

Below is the link to the electronic supplementary material.

Supplementary file1 (DOCX 45 KB)

## Data Availability

The datasets analysed during the current study are not publicly available due to institutional and privacy restrictions but are available from the corresponding author on reasonable request and subject to approval by the relevant ethics and data protection authorities.
