# Supplementary material for: Malnutrition Severity, Morphofunctional Assessment, and Survival in Patients with Lung Cancer: A Multicentre Prospective Cohort Study
Source: Lung. 2026 Aug 14;204(1):58. doi: 10.1007/s00408-026-00924-9 (PMC13473190; doi:10.1007/s00408-026-00924-9)
Supplement: Supplementary file 1 [file 408_2026_924_MOESM1_ESM.docx]

**SUPPLEMENTARY MATERIAL**

**Supplementary Table 1:** GLIM Diagnostic Framework: Phenotypic Criteria and Cut-off Values Used in the Study

**Supplementary Table 2**. Pairwise Comparisons of Clinical and Laboratory Characteristics Across GLIM Nutritional Categories

**Supplementary Table 3**. Pairwise Comparisons of Morphofunctional Parameters Across GLIM Nutritional Categories

**Supplementary Table 4**. Odds Ratios for Severe GLIM Status (Moderate vs Severe) According to BIVA, Ultrasound, Handgrip Strength, and Functional Performance

**Supplementary Table 5.** ECOG performance status according to GLIM-defined nutritional status and morphofunctional parameters

**Supplementary Table 6.** Missing data for variables included in the survival and morphofunctional analyses

**Supplementary Table 7.** Detailed Survival models

**Supplementary Table 1:** GLIM Diagnostic Framework: Phenotypic Criteria and Cut-off Values Used in the Study

| Tool | Description and formulas | Cut-off points | Categories | Reference |
| --- | --- | --- | --- | --- |
| Malnutrition screening | | | | |
| MNA-SF | 6-item questionnaire. Each question scored from 0 to 2 or 3. Total score range: 0–14 points. | 0-7 points | Malnutrition | *Rubenstein LZ, Harker JO, Salvà A, Guigoz Y, Vellas B. Screening for undernutrition in geriatric practice: developing the short-form mini-nutritional assessment (MNA-SF). J Gerontol A Biol Sci Med Sci. 2001 Jun;56(6):M366-72. doi: 10.1093/gerona/56.6.m366. PMID: 11382797.* |
|  |  | 8-11 points | Risk of malnutrition |  |
|  |  | 12-14 points | Normal nutrition status |  |
| Phenotypic criteria | | | | |
| Low Body Mass Index (BMI) | BMI = current weight (kg)/height(m²) | <70 years: <20 kg/m2  >70 years: <22 kg/m2 | Moderate GLIM severity | *Cederholm T, Jensen GL, Correia MITD, Gonzalez MC, Fukushima R, Higashiguchi T et al. GLIM criteria for the diagnosis of malnutrition - A consensus report from the global clinical nutrition community. Clin Nutr. 2019 Feb;38(1):1-9. doi: 10.1016/j.clnu.2018.08.002. Epub 2018 Sep 3. PMID: 30181091.* |
|  |  | <70 years: <18.5 kg/m2  >70 years: <20 kg/m2 | Severe GLIM severity |  |
|  |  | In the last 6 months: >10% Over more than 6 months: >20% | Severe GLIM severity |  |
| MNA= Mini Nutritional Assessment; BMI= Body Mass Index | | | | |

**Supplementary Table 2**. Pairwise Comparisons of Clinical and Laboratory Characteristics Across GLIM Nutritional Categories

| **Variables** | **Well vs Moderate** | **Well vs Severe** | **Moderate vs Severe** |
| --- | --- | --- | --- |
|  |  |  |  |
| Age (years) | 0.588 | 0.381 | 0.949 |
| Sex: | 0.288 | 0.795 | 0.473 |
| BMI, kg/m^2^ | <0.001 | <0.001 | 0.050 |
| HbA1c (%) | 0.867 | 0.817 | 0.602 |
| Total cholesterol (mg/dL) | 0.606 | 0.117 | 0.590 |
| Urea (mg/dL) | 0.829 | 0.973 | 0.804 |
| Creatinine (mg/dL) | 0.671 | 0.493 | 0.921 |
| Glomerular filtration (mL/min/1.73 m²) | 0.978 | 0.466 | 0.412 |
| Proteins (mg/dL) | 0.995 | 0.175 | 0.282 |
| Prealbumin (mg/dL) | 0.297 | 0.001 | 0.308 |
| Albumin (mg/dL) | 0.003 | 0.003 | 0.985 |
| C reactive protein (mg/dL) | 0.405 | 0.012 | 0.865 |
| TSH (μUI/mL) | 0.208 | 0.706 | 0.107 |
| ECOG | 0.002 | <0.001 | 0.007 |
| Cancer type | 0.591 | 0.270 | 0.591 |
| TNM | 0.499 | 0.007 | 0.213 |
| Chemotherapy | 1.000 | 0.022 | 0.045 |
| Radiotherapy | 0.393 | 0.393 | 1.000 |
| Immunotherapy | 0.865 | 0.030 | 0.121 |
| Complications | <0.001 | <0.001 | 0.433 |
| Exitus | 0.086 | <0.001 | 0.010 |
|  |  |  |  |

Data are represented as mean (SD) or n (%). Groups were divided according to the GLIM criteria, being well, moderate and severe the categories included in the study. A Shapiro-Wilks test was used to determine the normality of the variables. Asterisk indicates significant differences between groups according to the ANOVA or Kruskal test and further pairwise comparison of *p*-values, corrected for multiple comparisons test was used for variables expressed as percentage (****p*<0.001, ***p*<0.01, **p*<0.05). **Abbreviations**. BMI: Body Mass Index, GLIM: The Global Leadership Initiative on Malnutrition; HbA1c: Hemoglobin glycosylated; TSH: Thyroid-stimulating hormone

**Supplementary Table 3**. Pairwise Comparisons of Morphofunctional Parameters Across GLIM Nutritional Categories

| **Variables** | **Well vs Moderate** | **Well vs Severe** | **Moderate vs Severe** |
| --- | --- | --- | --- |
|  |  |  |  |
| **Anthropometric** |  |  |  |
| Weight, kg | <0.001 | 0.000 | 0.031 |
| Weight loss | 0.000 | <0.001 | <0.001 |
| **BIVA variables** |  |  |  |
| Xc | 0.663 | 0.632 | 0.310 |
| Rz | <0.001 | <0.001 | 0.810 |
| PA (º) | <0.001 | <0.001 | 0.043 |
| SPA | 0.548 | <0.001 | 0.008 |
| BCM, kg | <0.001 | <0.001 | 0.031 |
| FM, kg | <0.001 | <0.001 | 0.046 |
| FFMI, kg/m^2^ | <0.001 | <0.001 | 0.188 |
| FMI, kg/m^2^ | <0.001 | <0.001 | 0.062 |
| BCMI, kg/m^2^ | <0.001 | <0.001 | 0.029 |
| SMI, kg/m^2^ | <0.001 | <0.001 | 0.961 |
| MM, kg | 0.002 | <0.001 | 0.127 |
| ASMM, kg | <0.001 | <0.001 | 0.228 |
| FFM, kg | <0.001 | <0.001 | 0.141 |
| Hydragram®, % | 0.894 | 0.514 | 0.374 |
| Nutrigram®, mg/24h/m | <0.001 | <0.001 | 0.097 |
| Basal metabolism | 0.004 | <0.001 | 0.096 |
| TBW, kg | <0.001 | <0.001 | 0.276 |
| ECW, kg | 0.008 | 0.003 | 0.979 |
| Na/K | <0.001 | <0.001 | 0.001 |
| **Ultrasound** |  |  |  |
| RF-CSA, cm^2^ | <0.001 | <0.001 | 0.430 |
| RF-CIRC, cm | <0.001 | <0.001 | 0.484 |
| RF-X-axis, cm | 0.002 | 0.009 | 0.869 |
| RF-Y-axis, cm | <0.001 | <0.001 | 0.325 |
| L-SAT, cm | 0.032 | <0.001 | 0.271 |
| T-SAT, cm | 0.180 | <0.001 | 0.001 |
| S-SAT, cm | 0.633 | <0.001 | 0.038 |
| VAT, cm | <0.001 | <0.001 | 0.963 |
| GAT, cm | 0.003 | <0.001 | 0.012 |
| GATi, cm/m^2^ | 0.007 | <0.001 | 0.016 |
| **Functional variables** |  |  |  |
| Hand grip strength max, kg | 0.007 | <0.001 | 0.207 |
| Hand grip strength, kg | 0.005 | <0.001 | 0.177 |
| Up and Go, s | 0.041 | <0.001 | 0.026 |

Data are represented as mean (SD) or n (%). Groups were divided according to the GLIM criteria, being well, moderate and severe the categories included in the study. A Shapiro-Wilks test was used to determine the normality of the variables. Asterisk indicates significant differences between groups according to the ANOVA or Kruskal test and further pairwise comparison of *p*-values, corrected for multiple comparisons test was used for variables expressed as percentage (****p*<0.001, ***p*<0.01, **p*<0.05). **Abbreviations**. BCM: Body cell mass; BCMI: BCM index; BMI: Body mass index; BIVA: Bioelectrical Impedance Vectorial Analysis; FM: Fat mass; FMI: FM index; FFMI: Fat-free mass index; GLIM: The Global Leadership Initiative on Malnutrition; PA: Phase angle; RF-CIR: circumference of quadriceps rectus femoris; RF-CSA: rectus femoris cross-sectional area; SAT: subcutaneous adipose fat of leg (L), superficial (S) and total (T) abdominal; SMI: Skeletal muscle index; SPA: Standardized phase angle.

**Supplementary Table 4**. Odds Ratios for Severe GLIM Status (Moderate vs Severe) According to BIVA, Ultrasound, Handgrip Strength, and Functional Performance

| **Variables** | **OR (CI 95%)** | ***P* value** |
| --- | --- | --- |
|  |  |  |
| **BIVA variables** |  |  |
| PA (º) | 0.48 (0.3 - 0.73) | 0.001 |
| SPA | 0.68 (0.51 - 0.89) | 0.007 |
| BCM, kg | 0.83 (0.75 - 0.91) | 0.000 |
| FM, kg | 1.03 (0.95 - 1.11) | 0.529 |
| FFMI, kg/m^2^ | 0.79 (0.61 - 1.03) | 0.083 |
| FMI, kg/m^2^ | 1.21 (0.93 - 1.59) | 0.154 |
| BCMI, kg/m^2^ | 0.63 (0.47 - 0.83) | 0.001 |
| SMI, kg/m^2^ | 0.96 (0.72 - 1.29) | 0.792 |
| MM, kg | 0.93 (0.87 - 1) | 0.043 |
| ASMM, kg | 0.82 (0.7 - 0.95) | 0.013 |
| FFM, kg | 0.9 (0.84 - 0.97) | 0.007 |
| TBW, kg | 0.93 (0.85 - 1) | 0.067 |
| ECW, kg | 1.03 (0.93 - 1.15) | 0.565 |
| Na/K | 4.73 (1.14 - 23.73) | 0.047 |
| **Ultrasound** |  |  |
| RF-CSA, cm^2^ | 0.65 (0.46 - 0.88) | 0.008 |
| RF-CIRC, cm | 0.64 (0.47 - 0.86) | 0.003 |
| RF-X-axis, cm | 0.6 (0.36 - 0.95) | 0.040 |
| RF-Y-axis, cm | 0.39 (0.12 - 1.19) | 0.114 |
| L-SAT, cm | 0.82 (0.29 - 2.2) | 0.695 |
| T-SAT, cm | 0.5 (0.25 - 0.95) | 0.040 |
| S-SAT, cm | 0.44 (0.13 - 1.4) | 0.175 |
| VAT, cm | 0.56 (0.19 - 1.4) | 0.250 |
| GAT, cm | 0.65 (0.42 - 0.98) | 0.044 |
| **Functional variables** |  |  |
| Hand grip strength max, kg | 0.92 (0.88 - 0.96) | 0.001 |
| Hand grip strength, kg | 0.91 (0.86 - 0.95) | 0.000 |
| Up and Go, s | 1.25 (1.14 - 1.4) | 0.000 |
|  |  |  |

This table shows the results of logistic regression analysis assessing the association between GLIM nutritional status (**moderate** vs **severe**) and various clinical and functional variables. The dependent variable is GLIM status, with the **moderate** group as the reference and **severe** as the outcome of interest. Independent variables include measurements from bioelectrical impedance analysis (BIVA), ultrasound parameters, handgrip strength, and functional performance assessed by the **Timed Up and Go** test. For each variable, odds ratios (OR) with 95% confidence intervals (CI) and p-values are reported. The ORs represent the change in odds of being classified as severe GLIM per unit increase (or category) of the respective variable. An OR > 1 indicates higher odds of severe GLIM with increasing values, whereas an OR < 1 indicates lower odds. *p < 0.05, ** p < 0.01 and *** p < 0.001 considered statistically significant and is indicated by an asterisk () in the table **Abbreviations**. BCM: Body cell mass; BCMI: BCM index; BMI: Body mass index; BIVA: Bioelectrical Impedance Vectorial Analysis; FM: Fat mass; FMI: FM index; FFMI: Fat-free mass index; GLIM: The Global Leadership Initiative on Malnutrition; PA: Phase angle; RF-CIR: circumference of quadriceps rectus femoris; RF-CSA: rectus femoris cross-sectional area; SAT: subcutaneous adipose fat of leg (L), superficial (S) and total (T) abdominal; SMI: Skeletal muscle index; SPA: Standardized phase angle.

**Supplementary Table 5. Correlations between ECOG performance status and morphofunctional parameters**

| **Parameter** | **Spearman rho with ECOG-PS** | **P value** | **Direction** |
| --- | --- | --- | --- |
| Timed Up and Go | 0.432 | <0.001 | Higher values with worse ECOG-PS |
| Phase angle | -0.377 | <0.001 | Lower values with worse ECOG-PS |
| BCMI | -0.355 | <0.001 | Lower values with worse ECOG-PS |
| BCM | -0.334 | <0.001 | Lower values with worse ECOG-PS |
| RF-Y-axis | -0.314 | <0.001 | Lower values with worse ECOG-PS |
| RF-CSA | -0.283 | <0.001 | Lower values with worse ECOG-PS |
| FFMI | -0.257 | <0.001 | Lower values with worse ECOG-PS |
| FFM | -0.241 | <0.001 | Lower values with worse ECOG-PS |
| Calf circumference | -0.24 | <0.001 | Lower values with worse ECOG-PS |
| Handgrip strength max | -0.2 | <0.001 | Lower values with worse ECOG-PS |
| SMI | -0.195 | <0.001 | Lower values with worse ECOG-PS |
| Chair stand test, 5 repetitions | 0.161 | 0.012 | Higher values with worse ECOG-PS |

Data are shown as median (interquartile range), n (%), or Spearman correlation coefficient. ECOG-PS was compared across GLIM categories using the Kruskal-Wallis test, with pairwise Wilcoxon rank-sum tests and Holm correction. Correlations between ECOG-PS and morphofunctional parameters were assessed using Spearman correlation coefficients because ECOG-PS is an ordinal variable. BCM, body cell mass; BCMI, body cell mass index; ECOG-PS, Eastern Cooperative Oncology Group performance status; FFM, fat-free mass; FFMI, fat-free mass index; GLIM, Global Leadership Initiative on Malnutrition; IQR, interquartile range; RF-CSA, rectus femoris cross-sectional area; RF-Y-axis, rectus femoris anteroposterior diameter; SMI, skeletal muscle index.

| **Variable** | **Total cohort, n** | **Available, n** | **Missing, n** | **Missing, %** |
| --- | --- | --- | --- | --- |
| One-year follow-up / survival status | 374 | 357 | 17 | 4.5 |
| GLIM category | 374 | 374 | 0 | 0 |
| Age | 357 | 355 | 2 | 0.6 |
| Sex | 357 | 357 | 0 | 0 |
| BMI | 357 | 355 | 2 | 0.6 |
| ECOG performance status | 357 | 357 | 0 | 0 |
| BIA-derived markers | 357 | 355 | 2 | 0.6 |
| RF-CSA | 357 | 342 | 15 | 4.2 |
| RF-Y-axis | 357 | 338 | 19 | 5.3 |

**Supplementary Table 6. Missing data for variables included in the survival and morphofunctional analyses**

Data are shown for the main variables used in the survival and morphofunctional analyses. Missingness was low for the principal clinical covariates. Complete-case analysis was used for each model. BIA, bioelectrical impedance analysis; BMI, body mass index; ECOG, Eastern Cooperative Oncology Group; GLIM, Global Leadership Initiative on Malnutrition; RF-CSA, rectus femoris cross-sectional area; RF-Y-axis, rectus femoris anteroposterior diameter.

**Supplementary Table 7: Detailed survival models**

**A. Cox regression models for GLIM-defined nutritional status and one-year mortality**

| **Model** | **Variable** | **HR** | **95% CI** | **P value** |
| --- | --- | --- | --- | --- |
| Unadjusted | Moderate malnutrition vs well-nourished | 1.5 | 0.82-2.74 | 0.185 |
| Unadjusted | Severe malnutrition vs well-nourished | 4.31 | 2.73-6.80 | <0.001 |
| Adjusted | Moderate malnutrition vs well-nourished | 1.49 | 0.80-2.77 | 0.21 |
| Adjusted | Severe malnutrition vs well-nourished | 3.32 | 1.96-5.61 | <0.001 |
| Adjusted | Age | 0.99 | 0.97-1.01 | 0.523 |
| Adjusted | Male sex | 1.63 | 1.00-2.66 | 0.051 |
| Adjusted | BMI | 1 | 0.96-1.04 | 0.873 |
| Adjusted | ECOG-PS ≥2 | 2.17 | 1.34-3.51 | 0.002 |
| Adjusted | ECOG-PS unknown | 2.43 | 0.57-10.30 | 0.23 |

Adjusted model included age, sex, BMI, and ECOG performance status. Well-nourished patients were used as the reference category for GLIM-defined nutritional status. BMI, body mass index; CI, confidence interval; ECOG-PS, Eastern Cooperative Oncology Group performance status; GLIM, Global Leadership Initiative on Malnutrition; HR, hazard ratio.

**B. Proportional hazards assumption for GLIM Cox models**

| **Model** | **Term** | **Chi-square** | **P value** |
| --- | --- | --- | --- |
| GLIM unadjusted | GLIM category | 9.82 | 0.007 |
| GLIM unadjusted | Global | 9.82 | 0.007 |
| GLIM adjusted | GLIM category | 9.15 | 0.01 |
| GLIM adjusted | Age | 1.94 | 0.163 |
| GLIM adjusted | Sex | 0.19 | 0.665 |
| GLIM adjusted | BMI | 3.36 | 0.067 |
| GLIM adjusted | ECOG-PS | 3.8 | 0.15 |
| GLIM adjusted | Global | 13.43 | 0.062 |

The proportional hazards assumption was assessed using Schoenfeld residuals. The adjusted model included age, sex, BMI, and ECOG performance status.

**C. Cox models for morphofunctional cut-offs and one-year mortality**

| **Parameter** | **HR** | **95% CI** | **P value** |
| --- | --- | --- | --- |
| BCMI | 2.49 | 1.50-4.13 | <0.001 |
| Phase angle | 2.01 | 1.23-3.28 | 0.005 |
| BCM | 1.72 | 1.02-2.88 | 0.04 |
| RF-CSA | 1.31 | 0.80-2.14 | 0.289 |
| RF-Y-axis | 1.44 | 0.85-2.43 | 0.176 |
| FFMI | 1.78 | 1.01-3.14 | 0.046 |
| SMI | 1.58 | 0.98-2.57 | 0.063 |
| FFM | 1.77 | 1.04-3.02 | 0.035 |

Models were adjusted for age, sex, BMI, and ECOG performance status. HRs represent the mortality risk associated with values below the corresponding morphofunctional cut-off. BCM, body cell mass; BCMI, body cell mass index; FFM, fat-free mass; FFMI, fat-free mass index; RF-CSA, rectus femoris cross-sectional area; RF-Y-axis, rectus femoris anteroposterior diameter; SMI, skeletal muscle index.
